# Physiological and biochemical responses of selected weed and crop species to the plant-based bioherbicide WeedLock

**DOI:** 10.1038/s41598-022-24144-2

**Published:** 2022-11-15

**Authors:** Mahmudul Hasan, Anis Syahirah Mokhtar, Khairil Mahmud, Zulkarami Berahim, Adam Mustafa Rosli, Hafizuddin Hamdan, Mst. Motmainna, Muhammad Saiful Ahmad-Hamdani

**Affiliations:** 1 Department of Crop Science, Faculty of Agriculture, Universiti Putra Malaysia, 43400 Serdang, Selangor Malaysia; 2 Department of Plant Protection, Faculty of Agriculture, Universiti Putra Malaysia, 43400 Serdang, Selangor Malaysia; 3 Laboratory of Climate-Smart Food Crop Production, Institute of Tropical Agriculture and Food Security, Universiti Putra Malaysia, 43400 Serdang, Selangor Malaysia; 4 EntoGenex Industries Sdn. Bhd., Kuala Lumpur, Malaysia

**Keywords:** Biochemistry, Physiology, Plant sciences

## Abstract

WeedLock is a broad-spectrum plant-based bioherbicide that is currently on the market as a ready-to-use formulation. In this study, we investigated the physiological and biochemical effects of WeedLock (672.75 L ha^-1^) on *Ageratum conyzoides* L., *Eleusine indica* (L.) Gaertn, *Zea mays* L., and *Amaranthus gangeticus* L. at four different time points. WeedLock caused significant reductions in chlorophyll pigment content and disrupted photosynthetic processes in all test plants. The greatest inhibition in photosynthesis was recorded in *A. conyzoides* at 24 h post-treatment with a 74.88% inhibition. Plants treated with WeedLock showed increased malondialdehyde (MDA) and proline production, which is indicative of phytotoxic stress. Remarkably, MDA contents of all treated plants increased by more than 100% in comparison to untreated. The activity of the antioxidant enzymes superoxide dismutase (SOD), catalase (CAT), and peroxidase (POD) was elevated following treatment with WeedLock. Significant increases were observed in the SOD activity of *A. conyzoides* ranging from 69.66 to 118.24% from 6 to 72 h post-treatment. Our findings confirm that WeedLock disrupts the normal physiological and biochemical processes in plants following exposure and that its mode of action is associated with ROS (reactive oxygen species) production, similar to that of PPO (protoporphyrinogen oxidase) inhibitors, although specific site-of-action of this novel bioherbicide warrants further investigation.

## Introduction

Weeds are unwanted and troublesome plants growing abundantly in crop fields and gardens that can pose a serious hindrance to crop production by competing for growth resources such as nutrients and water, causing significant crop yield losses^1^. Weed population in crops field is influenced by the applied nutrients, especially nitrogen, so organic nutrient sources may help in weed management^2^. To reduce weed competition, many farmers across the continents opted for a time- and cost-efficient chemical weed control, including the adoption of herbicide-resistant crops varieties. Herbicide-tolerant plants provide the opportunity to control weeds by using herbicides as needed. Herbicide-tolerant cultivars of many crops have been developed by using the genetic diversity already present in the germplasm, making mutations, or through transgenic engineering^3–5^. Several different types of crops, including *Zea mays* L., *Triticum aestivum* L., *Oryza sativa* L., *Helianthus annus* L., *Glycine max* (L.) Merr., *Cicer arietinum* L., and *Medicago sativa* L., each have their unique genetic profile that determines their level of herbicide tolerance. Several potential mechanisms contribute to herbicide tolerance in germplasm or mutant lines, including modifications to the target enzyme's binding site, sequestration of herbicide molecule, improved herbicide metabolism, and overexpression of the target protein^6–8^.

Herbicides have been increasingly employed, especially in developing countries as the preferred method for controlling weeds. However, the frequent and intensive use of synthetic herbicides could contribute to increased environmental pollution, herbicide-resistant biotypes, residues in the food chain, health hazards and the deterioration of soil biological health^2,9^. Herbicide toxicity and duration of exposure are key factors in establishing the potential for adverse health effects. Even minimal toxicity can be dangerous if too much is inhaled, gets on the skin, or is consumed^10^. Minimizing herbicide use, choosing low-toxicity chemicals, and wearing protective equipment can reduce herbicide toxicity^11^.

In the wake of this, researchers have been working on developing environmentally friendly bioherbicide as an alternative to reduce the dangerous effects in agricultural production from overreliance on chemical herbicides. One such alternative developed in recent years is WeedLock. WeedLock is a plant-derived, broad-spectrum bioherbicide product that was developed and commercialized by EntoGenex Industries Sdn. Bhd. It is a contact herbicide that inhibits the plant’s ability to retain moisture and rigidity when absorbed by the aboveground parts of the plants. Weeds treated with WeedLock develop chlorosis and start to wither within an hour, and complete kill is normally achieved after a few days.

*Ageratum conyzoides* L., an annual weed from the Asteraceae family, is now a common weed in the tropical and subtropical regions and has widely spread across different habitats, wasteland areas, and roadsides^12^. *A. conyzoides* is a problematic weed for many crops and has caused significant yield reduction in seasonal crops^13^. *Eleusine indica* (L.) Gaertn is an annual grassy weed from the Poaceae family that is widely distributed in the tropics. As a noxious weed, it has been identified as a major problem in several crops where it causes severe yield losses^14,15^. In Malaysia, this problematic weed has evolved multiple resistance to glyphosate, glufosinate, paraquat, and group A (ACCase-inhibitors) herbicides, resulting in its control being rather challenging.

Various physiological and biochemical effects may be evoked in plants by herbicide exposure, including chlorosis, lipid peroxidation, and antioxidant responses^16^. Abiotic stress conditions strongly affect plants’ morphological, physiological, and biochemical activity, which leads to the overproduction of reactive oxygen species (ROS) and increased oxidative stress. Excessive ROS may cause lipid peroxidation and affect photosynthesis, resulting in cell injury and chlorosis^17^. ROS has been reported to be involved in abiotic and biotic stress signaling and response by influencing the expression of several genes and in turn affecting several processes like cell division, elongation, systemic signaling, and development^18^. Plant growth hormone maintains photosynthetic activity, antioxidant enzyme activation, and osmoprotectants accumulation in response to oxidative stress via inducing expression of stress-related genes^19,20^. Plant growth regulators including ethylene, salicylic acid, abscisic acid and jasmonic acid are known to promote growth and development of plants under optimal conditions. They have important roles during cell signaling and crosstalk as they provide tolerance to multiple stresses^21–23^. They may reduce herbicide exposure risk by modifying plant response to oxidative stress induced by herbicides.

Plants response to oxidative stress by enhancing their antioxidant defence systems, both enzymatic and non-enzymatic. The non-enzymatic system comprises chlorophyll, carotenoids, proteins, and amino acids etc., whereas superoxide dismutase (SOD), catalase (CAT), and peroxidase (POD) etc., constitute the enzymatic system^24^. SOD converts superoxide (O_2_^-^) to hydrogen peroxide (H_2_O_2_) in different cellular compartments, and H_2_O_2_ is eliminated by several enzymes such as POD and CAT^25^. Malondialdehyde (MDA) originates from the oxidation of unsaturated fatty acids regulated by ROS^26^. The amount of MDA in plants is a critical indicator of membrane damage owing to oxidative stress. Proline, a metal chelator and redox signaling molecule, has several functions during stress, such as regulating cytosolic acidity, scavenging free radicals, and promoting antioxidant activity, besides being involved in osmoprotection and osmotic adjustment^27^.

To date, numerous plant extracts have been identified with promising weed control properties, but little information has been reported on the mechanism or mode of action of these herbicides in plants that underlines the bioherbicide stress conditions. In this study, we investigated the biochemical and physiological changes caused by the bioherbicide WeedLock on selected weed and crop species, the understanding of which is imperative to developing an effective weed management strategy associated with this herbicide.

## Materials and methods

### Experimental location, treatments and design

The physiological and biochemical changes in test plants were assessed from July to September 2020, at Farm 15 of the Faculty of Agriculture, Universiti Putra Malaysia (3° 02' N latitude and 101° 42' E longitude at 31 m), Selangor, Malaysia. Two weeds namely *A. conyzoides* (voucher specimen#UPMWS001) and *E. indica* (voucher specimen#UPMWS027) and two crops *Z. mays* and *Amaranthus gangeticus* L. were used as test species in this experiment. The seeds of *Z*. *mays* and *A*. *gangeticus* were purchased from the Green World Genetics Sdn. Bhd., Rawang, Selangor, Malaysia. The collected seeds of test plants were identified and investigated in accordance with appropriate national and international policies. The weed seeds of *A*. *conyzoides* and *E*. *indica* were collected from Farm 15 at the Universiti Putra Malaysia. We collected the plant seeds with the proper permission of the institution’s authority by following the national and international strategies. Prior to executing the experiments, formal consent were obtained from the faculty’s farm management office.

Weed seeds were soaked in 0.2% potassium nitrate (KNO_3_) for 24 h, then rinsed with distilled water and grown in germination trays. Healthy, equal-size seedlings of the test plants were transplanted into plastic pots, one seedling in each pot (9 cm diameter). WeedLock was applied to the plants at 4–6-leaf stage for *A. conyzoides* and *A. gangeticus* and 2–3-leaf stage for *E. indica* and *Z. mays* at the application rate of 672.75 L ha^-1^ using a 1L multipurpose sprayer (Deluxe pressure sprayer), with a spray volume of 100 mL m^-2^. An adjustable hollow cone nozzle was used with 2 bar application pressure^28^. Mature leaves of each species were plucked between 8 and 10am, kept in aluminium foil, and brought to the laboratory from the glasshouse in an icebox. Then, the samples were frozen with liquid nitrogen and immediately stored at −80 °C. Samples for physiological analysis were collected at 3, 24, 48, and 72 h post-treatment, while samples for biochemical analysis were collected at 6, 24, 48, and 72 h post-treatment. Randomized complete block design (RCBD) was set with four replications for the experiment.. The factors in this experiment comprised different treatments and time points.

### Chlorophyll pigment content

Chlorophyll-*A*, Chlorophyll-*b*, and carotenoid contents were examined according to Lichenthaler and Bushman’s protocol^29^. Fresh leaves (0.1 g) were homogenized in 80% acetone (10 mL) in a glass bottle. The glass bottle was wrapped in aluminium foil and left in complete darkness for 72 to 96 h. When the incubation was finished, the leaf homogenate was transferred into a tube before it was vortexed and the sediment allowed to settle. The absorbance was measured at 663.2, 646.8, and 470 nm on a spectrophotometric reader. Acetone (80%) was used for the blank solution and chlorophyll content was expressed as mg g^-1^ of fresh weight using the following relationships:

1 $${\text{Chlorophyll-}}a\,\left( {\mu {\text{g ml}}^{{ - {1}}} } \right) = ({12}.{25} \times {\text{A663}}.{2} - {2}.{79} \times {\text{A646}}.{8})$$

2 $${\text{Chlorophyll-}}b\,\left( {\mu {\text{g ml}}^{{ - {1}}} } \right) = \left( {{21}.{5}0 \times {\text{A646}}.{8} - {5}.{1} \times {\text{A663}}.{2}} \right)$$

3 $${\text{Total chlorophyll }}\,(\mu {\text{g ml}}^{{ - {1}}} ) = ({7}.{15} \times {\text{A663}}.{2} + {18}.{71} \times {\text{A646}}.{8})$$

4 $$\text{Carotenoids} \,\left( {\mu\text{g ml}^{ - 1} } \right) = \frac{{\left( {1000 \times A470 - 1.8 \times \text{Chlorophyll a} - 85.02 \times \text{Chlorophyll b}} \right)}}{198}$$

### Photosynthetic rate, transpiration, and stomatal conductance

Between 9 and 11am, under sunlight, photosynthetic rate, transpiration, and stomatal conductance were measured for the leaves of the test plants using the LI-COR-6400XT Portable Photosynthetic System (Lincoln, Nebraska, U.S.A.). The measurements were taken on the abaxial surface at a CO_2_ flow rate of 400 μmol m^−2^ s^−1^, and the saturating photosynthetic photon flux density (PPFD) was 1000 mmol m^−2^ s^−1^^30^.

### Quantum yield (Fv/Fm)

Quantum yield was measured for the newest fully expanded leaf of test plants at 10-12am using a portable plant efficiency analyzer (Model FMS 2, Hansatech Instrument Ltd. UK)^31^.

### MDA content

Stewer and Bewley^32^ previously described the following methodology for determining malondialdehyde (MDA) content in the samples. Powdered leaf tissue (0.2 g) was homogenized in ultrapure distilled water (2 ml). Later, the samples were centrifuged (Sigma 3K30) for 15 min at 10,000 rpm. One milliliter of this solution was boiled in the water bath (90 °C; 30 min) along with a two-milliliter solution of trichloroacetic acid (TCA)/thiobarbituric acid (TBA) (Merck, German) and placed in an ice bath. The resulting mixture was again centrifuged at 10,000 rpm for 15 min. Finally, the supernatants were evaluated at three different wavelengths, namely 450, 532, and 600 nm, using a spectrophotometer to quantify the absorbance. MDA was expressed as µmol g^-1^ FW and determined using the following relationship:

5 $${\text{MDA }}\left( {\mu {\text{M}}} \right) = \{ {6}.{45} \times ({\text{A532}} - {\text{A6}}00) - 0.{56} \times {\text{A45}}0\}$$

where A450, A532, and A600 are the absorbance values at 450 nm, 532 nm, and 600 nm respectively.

### Proline content

Measurement of proline content was carried out according to the method reported by Bates et al.^33^, with minor modifications. Briefly, fresh leaves (0.1 g) of test plants were homogenized in 5% sulfosalicylic acid (2 ml). Test tubes with samples were centrifuged at 10,000 rpm for 10 min. One milliliter of the supernatant was mixed with 1 ml of glacial acetic acid and 1 ml of acid ninhydrin (1.25 g of ninhydrin; 30 ml of glacial acetic acid; 20 ml of phosphoric acid 6 M). The tubes with the solutions were incubated in a water bath (95 °C; 60 min) and then placed in an ice bath. Each tube was filled with 2 ml of toluene and then vortexed. The absorbance reading was determined at 520 nm while proline concentration was calculated using the standard L-Proline value (Sigma-Aldrich USA). The concentration of proline was calculated using the following equation;

6 $${\text{Proline}} \left({\upmu{\rm mol\, g}^{ - 1} {\rm FW}} \right) =\frac{\frac{{{\text{Proline}}} \left( {\upmu \,{\rm g \,ml}^{ - 1} } \right)\times {\rm Tolune}\,\left( {\rm ml} \right)}{{{{115.5 \left( {\upmu{\rm g}\, \upmu {\rm mole}^{- 1} } \right)}}}}}{{\rm Fresh \,weight \,of \,sample} \left( {\rm g} \right)}$$

where 115.5 (μg μmole^-1^) is the molecular weight of proline.

### Extraction of enzymes

Enzyme extraction was performed according to the procedure described by Gupta et al.^34^. In a porcelain mortar, fresh leaves of the test plants were ground to a fine powder in liquid nitrogen. After that, 0.1 g of the powdered sample was mixed with potassium phosphate buffer (1.5 ml) and centrifuged at 10,000 rpm for 20 min.

### SOD activity

The procedure reported by Gupta et al.^34^ was followed to prepare the reaction mixture composed of the following components: enzyme extract (0.05 ml), 100 mM potassium phosphate buffer (1.5 ml), 3 mM EDTA (0.1 ml), 200 mM methionine (0.1 ml), 2.25 mM NBT (n-nitro blue tetrazolium) (0.01 ml), and ultra-pure distilled water (1 ml). In the dark, 60 μM riboflavin was added to the reaction mixture. The tubes were then incubated for 10 min under a 15-W fluorescent bulb. The control mixture was kept in the light and did not contain any enzyme extract. The blanks were reaction mixtures which did not contain any enzyme extract and were not kept under the light to immediately stop the reaction after incubation. The tubes were covered in aluminium foil. Superoxide dismutase (SOD) activity was measured using a spectrophotometer (UV-3101PC) by recording absorbance changes at 560 nm for 1 min caused by the reaction of the superoxide nitroblue tetrazolium complex with the enzyme extract. Each unit of enzyme production was measured based on the amount of enzyme that caused 50% reduction of NBT. SOD activity was expressed as g^-1^ FW, as follows:

7 $${\text{SOD }}\left( {{\text{\% inhibition}}} \right) = \frac{{\left( {{\text{A}}560{\text{ control}} - {\text{A}}560{\text{ sample}}} \right) \times 100}}{{{\text{A}}560{\text{ control}}}}$$

8 $${\text{SOD }}\left( {{\text{unit ml}}^{ - 1} } \right) = \frac{{{\text{\% inhibition}} \times {\text{total volume}}}}{{50 \times {\text{enzyme volume}}}}$$

9 $${\text{SOD }}\left( {{\text{unit mg}}^{ - 1} {\text{ FW}}} \right) = \frac{{{\text{unit mL}}^{ - 1} }}{{{\text{enzyme }}\left( {{\text{mg mL}}^{ - 1} } \right)}}$$

### POD activity

Peroxidase (POD) activity was determined using the following equations according to Rao et al.^35^ and evaluated at 470 nm with guaiacol oxidation by H_2_O_2_. Enzyme extract (0.1 ml), 10 mM phosphate buffer (2.83 ml; pH 7.0), and 20 mM guaiacol (0.05 ml) were mixed in a 3 ml reaction mixture (pH 7.0). The reaction was started by adding 0.02 ml of 40 mM H_2_O_2_. The mixture that did not contain an enzyme extract was referred to as ‘blank’. The blank tube was placed in a spectrophotometer for 4 to 5 min in order to achieve temperature equilibrium. POD activity was expressed as μmol min^-1^ g^-1^ FW.

10 $$\text{POD} \left( {{\upmu} \text{mol}\, \text{min}^{ - 1} ml^{ - 1} } \right) = \frac{{\left( {A470/\text{min} } \right) \times \text{total volume} \times 1000}}{26.6 \times \text{enzyme volume}}$$

11 $$\text{POD} \left( {{\upmu}\text{mol}\, \text{min}^{ - 1} \text{mg}^{ - 1} FW} \right) = \frac{{\text{unit}\, mL^{ - 1} }}{{\text{enzyme} \left( {\text {mg ml}^{ - 1} } \right)}}$$

The wavelength for absorption reading was 470 nm for 1 min and the extinction coefficient was 26.6 mM^-1^ cm^-1^.

### CAT activity

Catalase (CAT) activity was measured based on the procedure described by Aebi^36^, as shown in the equations below. A 3 ml reaction mixture was prepared, comprising 100 mM potassium phosphate buffer (1.5 ml), 75 mM H_2_O_2_ (0.5 ml), enzyme extract (0.05 ml), and ultrapure distilled water (0.95 ml). The mixture that did not contain any enzyme extract was referred to as ‘blank’ and was placed in a spectrophotometer for 4 to 5 min to achieve temperature equilibration. The reading of the absorption wavelength was carried out in the spectrophotometer for 2 min. CAT activity was determined by measuring the amount of enzyme that decomposed 1 μM of H_2_O_2_ and was expressed as μmol min^-1^ g^-1^ FW.

12 $$ \text{CAT} \left( {{\upmu} \text{mol}\, \text{min}^{ - 1} ml^{ - 1} } \right) = \frac{{\left( {A240/\text{min} } \right) \times \text{total volume} \times 1000}}{43.6 \times \text{enzyme volume}}$$

13 $$\text{CAT} \left( {{\upmu} \text{mol} \text{min}^{ - 1} mg^{ - 1} FW} \right) = \frac{{\mu \text{mol} \text{min}^{ - 1} ml ^{ - 1} }}{{\text{enzyme} \left( {mg ml^{ - 1} } \right)}}$$

The wavelength for absorption reading was 240 nm for 1 min, and the extinction coefficient was 43.6 M^-1^ cm^-1^.

### Statistical analysis

Data analysis was carried out using a two-way ANOVA on SAS (statistical analysis system) software version 9.4 (Cary, NC, USA) to determine significant differences between treatment and untreated groups at different time points. The effects of replication on studied parameters were not significant. Tukey test was used to compare means at a 0.05% probability level.

## Results

### Effect of WeedLock on the photosynthesis, transpiration, stomatal conductance, and maximum quantum yield of test plants

Photosynthesis, transpiration, stomatal conductance, and maximum quantum yield of *A. conyzoides*, *E. indica*, *Z. mays*, and *A. gangeticus* were significantly (*p* ≤ 0.05) affected by the application of WeedLock at different time points (Table 1). Significant difference was recorded between treatments and time points for the observed parameters of the test plants. The greatest inhibition in photosynthetic rate by WeedLock was recorded in *E. indica* (25.44%), followed by *A. conyzoides* (24.06%), *A. gangeticus* (23.23%), and *Z. mays* (20.61%), as compared to untreated at 3 h post-treatment. At 24 h post-treatment, all test plants recorded significant differences as compared to untreated except for *A. conyzoides*. Stomatal conductance of treated plants did not differ significantly at 24, 48, and 72 h post-treatment between time points. At 3 h post-treatment, the inhibition rates recorded were 23.45%, 21.48%, 24.47%, and 25.47% for *A. conyzoides*, *E. indica*, *Z. mays*, and *A. gangeticus* respectively.

**Table 1:** Effect of WeedLock on physiological processes of test plants.

Test plants	Treatments	Photosynthesis rate (µmol m^-2^ s^-1^)					Stomatal conductance (mol m^-2^ s^-1^)					Transpiration rate (mmol m^-2^ s^-1^)					FV/FM				
		3 h	24 h	48 h	72 h	HSD	3 h	24 h	48 h	72 h	HSD	3 h	24 h	48 h	72 h	HSD	3 h	24 h	48 h	72 h	HSD
*A. conyzoides*	Untreated	17.68a	16.28b	16.60ab	16.41b	1.13	0.40a	0.36a	0.38a	0.35a	0.07	7.31a	6.21b	7.21ab	7.10ab	1.05	0.80a	0.83a	0.80a	0.81a	0.07
	WeedLock	13.42a	4.09b	3.62b	3.60b	0.86	0.31a	0.11b	0.13b	0.10b	0.04	5.58a	1.41b	1.56b	1.52b	0.36	0.57a	0.20b	0.17b	0.18b	0.08
*E. indica*	Untreated	27.60a	25.14ab	26.21ab	24.73b	2.80	0.27a	0.25a	0.26a	0.24a	0.04	8.55a	6.81b	7.36ab	7.12b	1.38	0.74a	0.76a	0.75a	0.78a	0.08
	WeedLock	20.42a	6.79b	6.61b	5.98b	1.37	0.21a	0.12b	0.11b	0.10b	0.03	6.30a	1.68b	1.79b	1.69b	0.91	0.53a	0.20b	0.18b	0.17b	0.08
*Z. mays*	Untreated	16.87a	14.88a	14.56a	16.65a	2.33	0.29a	0.27ab	0.23b	0.28a	0.04	4.65a	4.24a	4.17a	4.45a	0.60	0.77a	0.81a	0.77a	0.79a	0.07
	WeedLock	13.36a	4.73b	4.24b	4.73b	0.80	0.22a	0.14b	0.12b	0.11b	0.04	3.48a	1.21b	1.17b	1.13b	0.36	0.58a	0.34b	0.32b	0.35b	0.13
*A. gangeticus*	Untreated	12.34a	11.80ab	10.12b	11.20ab	1.69	0.30a	0.27a	0.23a	0.25a	0.10	3.38a	3.33a	3.28a	3.30a	0.19	0.79a	0.80a	0.74a	0.78a	0.06
	WeedLock	9.46a	3.44b	2.77b	2.94b	0.81	0.23a	0.11b	0.10b	0.09b	0.07	2.48a	0.85b	0.77b	0.75b	0.19	0.58a	0.23b	0.19b	0.21b	0.11

Means having different letters differ significantly at *p* < 0.05.

The inhibition of transpiration rate from WeedLock exposure started showing significant differences at 24 h following treatment, and the highest inhibition was recorded for all test plants at 72 h post-treatment. At 48 h following treatment, 78.31%, 76.52%, 75.68%, and 71.96% of inhibition were recorded in *A. conyzoides*, *A. gangeticus*, *E. indica*, and *Z. mays*, respectively, as compared to untreated. Although significant differences were not observed between time points from 24 to 72 h post-treatment, inhibition of transpiration was still observed between treatment and untreated groups across the time points. WeedLock treatment negatively affected the Fv/Fm ratio of the test plants across the different time points and resulted in a progressive reduction in the quantum yield of photosystem II (PS II). The results show declines ranging from 28.67 to 77.56%, 27.78 to 77.67%, 25.32 to 56.22%, and 27.17 to 73.30% from 3 to 72 h following WeedLock application in *A. conyzoides*, *E*. *indica*, *Z. mays*, and *A. gangeticus* respectively, as compared to untreated. *Z. mays* recorded only a 58.02% decline after 24 h, which was the lowest inhibition relative to *A. conyzoides*, *E. indica,* and *A. gangeticus* in response to WeedLock exposure. At 48 h, *A. conyzoides* recorded the greatest reduction of 78.25% in Fv/Fm ratio, followed by *E. indica* (75.88%), *A. gangeticus* (73.92%), and *Z. mays* (58.77%).

### Effect of WeedLock on the chlorophyll pigments of test plants

Marked declines in chlorophyll pigment content were observed in *A. conyzoides*, *E. indica*, *Z. mays,* and *A. gangeticus* following WeedLock treatment at different time intervals (Table 2). Significant differences were recorded across treatments and different time points in test plants post-treatment. At 3 h after exposure, the chlorophyll-*a* contents of *A. conyzoides, E. indica*, *Z. mays*, and *A. gangeticus* were reduced by 33.64%, 30.03%, 25.82%, and 29.64% respectively by WeedLock treatment, as compared to untreated. No significant difference was observed in chlorophyll-*a* content in test plants between 24, 48, and 72 h following treatment. For all the time points observed, the reduction of chlorophyll-*a* content was relatively higher in *A. conyzoides,* as compared to *E. indica*, *Z. mays*, and *A. gangeticus*.

**Table 2:** Effect of WeedLock on pigment content of test plants.

Test plants	Treatments	Chlorophyll-*a* (mg g^-1^ FW)					Chlorophyll-*b* (mg g^-1^ FW)					Total Chlorophyll (mg g^-1^ FW)					Carotenoids (mg g^-1^ FW)				
		3 h	24 h	48 h	72 h	HSD	3 h	24 h	48 h	72 h	HSD	3 h	24 h	48 h	72 h	HSD	3 h	24 h	48 h	72 h	HSD
*A. conyzoides*	Untreated	3.32a	3.21a	3.24a	3.20a	0.20	1.35a	1.30a	1.32a	1.29a	0.26	4.67a	4.52a	4.56a	4.49a	0.18	0.96a	0.94a	0.95a	0.93a	0.09
	WeedLock	2.20a	0.80b	0.74b	0.74b	0.19	0.97a	0.52b	0.49b	0.47b	0.20	3.18a	1.32b	1.23b	1.20b	0.18	0.66a	0.31b	0.28b	0.27b	0.07
*E. indica*	Untreated	2.87a	2.75a	2.72a	2.70a	0.20	1.18a	1.15a	1.10a	1.07a	0.39	4.06a	3.90a	3.82a	3.77a	0.44	0.88a	0.86a	0.85a	0.86a	0.08
	WeedLock	2.01a	0.70b	0.68b	0.72b	0.14	0.88a	0.44b	0.38b	0.44b	0.20	2.89a	1.15b	1.06b	1.16b	0.21	0.63a	0.27b	0.26b	0.29b	0.07
*Z. mays*	Untreated	2.98a	2.95a	2.94a	2.90a	0.18	1.42a	1.40a	1.35a	1.30a	0.27	4.40a	4.35a	4.29a	4.21a	0.21	0.92a	0.90a	0.88a	0.87a	0.09
	WeedLock	2.21a	0.99b	0.89b	0.93b	0.20	1.13a	0.64b	0.55b	0.51b	0.24	3.34a	1.63b	1.45b	1.44b	0.24	0.70a	0.35b	0.34b	0.31b	0.09
*A. gangeticus*	Untreated	3.25a	3.18a	3.20a	3.15a	0.17	1.32a	1.26a	1.29a	1.25a	0.23	4.57a	4.45a	4.49a	4.41a	0.31	0.97a	0.92a	0.94a	0.90a	0.07
	WeedLock	2.28a	0.94b	0.81b	0.90b	0.24	0.96a	0.53b	0.51b	0.53b	0.31	3.25a	1.47b	1.32b	1.42b	0.28	0.65a	0.34b	0.30b	0.35b	0.08

Means having different letters differ significantly at *p* < 0.05.

The reduction in chlorophyll-*b* content ranged from 27.44 to 63.58% for *A. conyzoides*, 24.13 to 58.22% for *E. indica*, 20.75 to 60.71% for *Z. mays*, and 26.05 to 58.00% for *A. gangeticus* from 3 to 72 h post-treatment. At 48 h post-treatment, WeedLock exerted similar effects on all test plants where reductions were observed: *A. conyzoides* (62.65%), *E. indica* (61.51%), *A. gangeticus* (59.98%), and *Z. mays* (59.16%). At 72 h post-treatment, chlorophyll-*b* content reduction was lowest in *A. gangeticus* (58.00%) and highest in *A. conyzoides* (63.58%).

WeedLock reduced the total chlorophyll contents of all test plants across all time points, with significant differences recorded from 24 to 72 h post-treatment. Total chlorophyll content of test plants differed significantly (*p* ≤ 0.05) from 3 to 24 h following treatment, though no significant difference was recorded between the later time points of 24, 48, and 72 h. Carotenoid content was significantly lower in all WeedLock-treated plants in comparison to untreated from 24 to 72 h post-treatment. Similar levels of reduction were observed among the test plants across the different time points; at 24 h post-treatment, *A. conyzoides* recorded the highest reduction of 66.17%, followed by *E. indica* of 65.11%, *A. gangeticus* of 63.18%, and *Z. mays* of 60.69%. At 48 h post-treatment, the lowest reduction following WeedLock application was recorded in *Z. mays* (61.56%) relative to the other species. At 72 h post-treatment, carotenoid content reduction was highest in *A. conyzoides* a 70.41% inhibition whereas the lowest reduction of 61.90% was observed in *A. gangeticus.*

### Effect of WeedLock on the proline and MDA content of test plants

WeedLock caused proline and MDA contents to increase for all time points, with significant differences (*p* ≤ 0.05) observed across all species at 6 h post-treatment (Table 3). Proline and MDA content recorded significant differences across treatments and different time points post-treatment. The increase in proline content was relatively higher in *A. conyzoides* as compared to *E. indica*, *Z. mays*, and *A. gangeticus* at all time points. At 6 h following exposure, *A. conyzoides* exhibited the highest increase of 91.05% as compared to the other species, where significant differences were observed for all species as compared to untreated. This increasing trend continues across later time points from 24 to 72 h, though significant differences were not observed for all species as compared to untreated. At 6 h, MDA content increased significantly in all test species following WeedLock treatment, and this trend continued from 6 to 72 h for all species except for *E. indica*. Also, at 6 h, the MDA content of *A. conyzoides* (58.90%) was markedly higher than that of *E. indica* (56.17%), *Z. mays* (46.00%), and *A. gangeticus* (50.57%). At 24 h post-treatment, the MDA contents of all treated plants increased by over 100% following treatment, and similar trends were observed at 48 h and 72 h. The differences in MDA content between treated and untreated plants showed an increasing trend across time points from 6 to 72 h.

**Table 3:** Effect of WeedLock on proline and MDA content of test plants.

Test plants	Treatments	Proline (µmol g^-1^ FW)					MDA (µmol g^-1^ FW)				
		6 h	24 h	48 h	72 h	HSD	6 h	24 h	48 h	72 h	HSD
*A. conyzoides*	Untreated	5.48a	5.55a	5.34a	5.44a	0.38	3.59a	3.25b	3.37ab	3.53ab	0.28
	WeedLock	10.48b	14.07a	14.13a	14.65a	0.83	5.70d	7.12c	7.77b	8.23a	0.25
*E. indica*	Untreated	4.37a	4.16a	4.24a	4.18a	0.31	2.36a	2.25a	2.12a	2.21a	0.36
	WeedLock	8.15c	10.24b	10.90ab	10.95a	0.70	3.68b	4.86a	4.81a	5.12a	0.76
*Z. mays*	Untreated	5.09a	4.91a	5.03a	4.96a	0.29	2.28a	2.20a	2.22a	2.24a	0.14
	WeedLock	8.77c	11.54b	12.16a	12.19a	0.61	3.33d	4.47c	4.81b	5.00a	0.18
*A. gangeticus*	Untreated	5.69a	5.34b	5.52ab	5.45ab	0.32	3.34a	2.97a	3.07a	3.15a	0.60
	WeedLock	10.21c	13.06b	13.88a	14.09a	0.74	4.99c	6.22b	6.67ab	7.02a	0.74

Means having different letters differ significantly at *p* < 0.05.

### Effect of WeedLock on antioxidant enzyme activity in test plants

In general, WeedLock increased the antioxidant enzyme activity of *A. conyzoides*, *E. indica*, *Z. mays*, and *A. gangeticus* across all time points, represented by the SOD, POD, and CAT enzymes (Table 4). Significant differences were observed for all test species in SOD activity at 6 h and 24 h post-treatment, and in POD and CAT activity at 6 h post-treatment only. The increases in SOD activity observed in *A. conyzoides* ranged from 69.66% to 118.24% from 6 to 72 h following treatment, as compared to untreated. At 72 h post-treatment, SOD activity in *A.* *conyzoides* was highest with a 131.54% increase, and lowest in *Z. mays* with a 105.28% increase. Similarly, POD activity of test plants increased considerably across all time points in comparison to untreated. POD activity in *A. conyzoides* was 117.04% higher than untreated at 24 h following treatment. Significant differences were recorded between 6 and 24 h in WeedLock-treated plants, but not for later time points from 24 to 72 h. The different plant species showed similar trends of increased CAT activity across all time points following treatment with WeedLock. At 6 h post-treatment, irrespective of the species, significant increases were recorded in CAT activity as compared to untreated. Also, at 6 h, the CAT activity of *A. conyzoides* showed the highest increase of 34.93%, followed by *E. indica* (30.55%), *A. gangeticus* (29.20%), and *Z. mays* (27.05%), as compared to untreated. At 24 h following treatment, a 43.59% increase in CAT activity was observed in *Z. mays*, which was the lowest increase as compared to *A. conyzoides*, *E. indica,* and *A. gangeticus*. At 72 h, *A. conyzoides* showed the highest increase of 65.39% in CAT activity following WeedLock treatment, while *Z. mays* recorded the lowest increase of 47.18%.

**Table 4:** Effect of WeedLock on antioxidant enzyme activity of test plants.

Test plants	Treatments	SOD (Unit g^-1^ FW)					POD (µmol g^-1^ FW)					CAT (µmol g^-1^ FW)				
		6 h	24 h	48 h	72 h	HSD	6 h	24 h	48 h	72 h	HSD	6 h	24 h	48 h	72 h	HSD
*A. conyzoides*	Untreated	4.81a	4.41a	4.56a	4.75a	1.47	10.71a	12.41a	11.56a	12.97a	3.11	5.40a	5.19a	5.30a	5.16a	0.57
	WeedLock	7.52b	9.14ab	9.76ab	11.01a	3.23	18.61b	26.79a	25.66a	29.32a	6.13	7.57b	8.39ab	8.70a	8.53a	0.92
*E. indica*	Untreated	4.12a	3.67a	3.78a	3.84a	0.65	18.89a	21.43a	21.71a	20.58a	4.00	5.06a	4.92a	4.78a	4.82a	0.62
	WeedLock	6.51b	7.67ab	8.11a	8.31a	1.30	33.83b	48.21a	50.47a	49.06a	8.44	6.60b	7.53a	7.46a	7.67a	0.84
*Z. mays*	Untreated	4.12a	3.80a	3.99a	4.14a	1.05	16.92a	18.61a	18.33a	18.89a	3.38	5.09a	5.06a	4.82a	4.75a	0.44
	WeedLock	5.92b	7.60ab	8.09ab	8.50a	2.41	26.79b	39.19a	39.76a	41.16a	6.67	6.64b	7.26a	7.12a	6.98ab	0.43
*A. gangeticus*	Untreated	4.64a	4.57a	4.43a	4.65a	1.39	11.84a	12.12a	12.69a	12.41a	3.90	5.92a	5.68a	5.54a	5.47a	0.51
	WeedLock	7.02b	9.26ab	9.30ab	9.86a	2.83	18.61b	25.94ab	27.91a	27.90a	8.21	7.64b	8.67a	8.61a	8.77a	0.80

Means having different letters differ significantly at *p* < 0.05.

## Discussion

Excessive use of synthetic herbicides can lead to an increased number of herbicide resistant biotypes, low agricultural productivity, environmental pollution, as well as serious health hazards. Concerns over these issues have prompted the interest in exploring alternative weed management strategies using natural products for better sustainability^37,38^. Our previous work has demonstrated that WeedLock is a promising novel bioherbicide with an excellent weed control efficacy under both glasshouse and field conditions^39^. In this study, the physiological and biochemical changes in *A. conyzoides*, *E. indica*, *Z. mays*, and *A. gangeticus* following WeedLock exposure at different time points were investigated as a continuation to our previous work.

Based on our findings, photosynthesis was inhibited in all test species following exposure to WeedLock across all time points, with the highest reduction recorded in *A. conyzoides.* Reduction in photosynthesis results in oxidative stress and increased intracellular ROS production, damaged macromolecules, and lower plant defence levels^40^. Plant growth is significantly affected by oxidative stress in adverse environmental conditions, and perturbations to photosynthesis are the primary source of oxidative stress. Stomatal conductance of the test plants was remarkably inhibited at 24 h and later by the application of WeedLock. The mechanism of the closing and opening of the stomata is a crucial feature in plants that helps in minimizing water loss, thereby influencing gas exchanges. Transpiration rates of test plants were also negatively affected by WeedLock treatment across the different time points, with significant results recorded at 48 h for all plant species. Stomatal conductance is known to be associated with decreased photosynthesis and transpiration, both of which may be influenced by stress. During a plant’s stomatal opening, water is evaporated by transpiration, and carbon dioxide (CO_2_) is taken in through photosynthesis^41^. The findings from our study are consistent with those of Zohaib et al.^42^, who reported the inhibitory effect of *Vicia sativa* L., *Trigonella polycerata* M. Bieb., *Medicago polymorpha* L., and *Lathyrus aphaca* L. extracts on the photosynthesis of *O. sativa*.

Chlorophyll fluorescence is a powerful feature in plants for studying the effect of stresses on the photosynthetic process, which is indicative of the maximum quantum yield of PSII (Fv/Fm)^43^. Our results revealed that maximum quantum yield was significantly affected in test plants by treatment with WeedLock at 24 h and later, where the percentage of damage was relatively higher in *A. conyzoides*. Thylakoid membrane damage and inhibition of energy transfer from antenna molecules to reduction centres can lead to photo-inhibition damage and lower the Fv/Fm ratio in plants^44^. This may consequently lead to the inhibition of the primary reduction of photosynthesis and ultimately hinder the production of photosynthetic products.

Our findings indicated that, regardless of plant species, chlorophyll pigments were highly sensitive to WeedLock. In plants, chlorophyll pigments play an important role in the photosynthesis process and serve as a green pigment embedded in photosynthetic membranes, resulting in a reduction in chlorophyll pigment normally leads to a reduction in photosynthesis. In this study, treatment groups showed chlorotic and leaf blight symptoms on the test plants, leading to wilting and disrupted growth. This chlorotic and delay in growth may be attributed to the phytotoxic properties of WeedLock. Reduced chlorophyll content may be a result of the allelochemicals in plant extracts interfering with thylakoid membranes or chlorophyll biosynthesis, or inhibiting the enzymes involved in chlorophyll biosynthesis. Pigment content is involved in the chlorophyll biosynthesis pathway, and allelochemicals distort photosynthetic efficiency^45^. A higher level of the chlorophyllase enzyme is associated with a lower chlorophyll content when the plant is subjected to stressful conditions^17^. Carotenoids acted as an antioxidant, protecting cells from damage caused by free radicals and photochemical reactions^46^. *Portulaca oleracea* L. root extract led to a decrease in chlorophyll b and carotenoids of 81.40 and 77.8%, respectively; this may have resulted from a decrease in chlorophyll biosynthesis or degradation of existing chlorophyll^45^. Several reports have shown that plant extracts cause inhibition in chlorophyll and inhibit photosynthetic processes in plants^17,47^.

In this work, the highest increases in proline (169.27%) and MDA (133.47%) contents following treatment with WeedLock were observed in *A. conyzoides*, as compared to those in *E. indica*, *Z. mays,* and *A. gangeticus*. These results of increased proline content were expected, which reflects the leaf damage and stress which was detected during the study. Proline accumulation in plant leaves in response to stress may help stabilize protein molecules and membranes as a defence against WeedLock-induced stresses. In a similar case, the application of *Haloxylon persicum* Bunge. water extract significantly increased the proline contents of *T. aestivum* and *Brassica nigra* (L.) K.Koch^48^. The noticeably higher increase in the amount of MDA in *A. conyzoides* compared to other test plants indicates *A*. *conyzoides* showed more sensitivity to the cell damage induced by WeedLock, resulting in a greater growth reduction of the plant. Oxidative damage and ROS synthesis are triggered by the stress caused by allelochemicals, which leads to subcellular structural disruption. Ullah et al.^49^ reported similar results in which the MDA content of *T. aestivum* and *B. napus* also increased following treatment with *Phytolacca latbenia* (Moq.) H. Walter methanolic extract.

The phytotoxic effects of WeedLock observed in treated plants might be associated with ROS regulation in the leaf tissue. As shown in this study, the activity of plant defence enzymes such as SOD, POD, and CAT increased significantly in *A. conyzoides*, *E. indica*, *Z. mays,* and *A. gangeticus* following WeedLock treatment. The growth inhibition observed in treated plants is indicative of the phytotoxic effects of WeedLock which interferes with the leaf tissue system. Similarly, previous studies also reported an increased activity in SOD, POD, and CAT in the treated plants following treatment with plant extracts bioherbicide^50,51^. In addition, the results suggest that WeedLock may trigger lipid peroxidation in plants, by which they are subjected to oxidative damage. Following exposure to WeedLock, the plant may gradually produce ROS in the leaves that hinders the ability of antioxidant enzymes to mitigate oxidative stress. Generation of ROS is an important process shared both by abiotic and biotic stress responses in plants^17^ which mainly occurs in the mitochondria, peroxisome, chloroplast, and plasma membrane. Although ROS are highly oxidative, they also play an essential role in regulating many cell processes, including the production of plant stress hormones (salicylic acid, jasmonic acid, ethylene), programmed cell death, stomatal behaviour, and hormonal signaling. Cross-talk between salicylic acid and other phytohormones or different signaling molecules in both normal and stress conditions was also evident^52,53^. Salicylic acid potentially controls, either directly or indirectly, the activity of antioxidant defence enzymes and alters plant responses to multiple stress^52,53^, including those caused by herbicides^54,55^. Both phytohormone-ethylene and gibberellin serve important roles in mitigating stress by activating defence regulatory genes^56^. Several genes were associated with gibberellin and phytoalexin production and Mitogen activated protein kinase signaling were downregulated by caffeic acid, resulting in growth inhibition of *Setaria viridis* (L.) P.Beauv^57^.

Our current findings suggest that the plausible mode of action of WeedLock and other plant extract bioherbicides could be through ROS generation and ROS-induced cell deaths, albeit determining specific pathways and enzymes employed by the bioherbicide warrants further investigation. Nevertheless, it is established that WeedLock enters the plant tissue through the cell membrane and alters the activity and function of certain enzymes, resulting in the observed phytotoxic effects in plants. WeedLock is a non-selective contact herbicide that can control a wide range of weed species and has the potential to be used as a bioherbicide for sustainable weed management in both cropping and non-cropping zones.

## Conclusion

The physiological and biochemical responses evoked in plants treated with the bioherbicide WeedLock indicate the manifestation of phytotoxic stress in test plants.. The findings from this study show that WeedLock significantly affected the photosynthesis and antioxidant enzyme activity of the test plants with the following order of phytotoxicity, from highest to lowest: *A. conyzoides* > *E. indica* > *A. gangeticus* > *Z. mays*. This situation offers the opportunity to control weeds that are resistant to the present herbicides. In addition, specific target site are needed to investigate through molecular mechanisms, metabolomics profiling, proteomics, modern and sophisticated methods of chemistry and biochemistry.

## Supplementary Information

Supplementary Information.

## Data Availability

All data generated or analysed during this study are included in this article [and its supplementary information files].
